# Invited Commentary: Nationwide Study on Stress Perception Among Surgical Residents

**DOI:** 10.1007/s00268-022-06560-7

**Published:** 2022-04-16

**Authors:** R. Marijn Houwert, Menno R. Vriens

**Affiliations:** Department of Surgery, University Medical Center Utrecht, Utrecht, The Netherlands

We congratulate colleagues Guglielmetti et all for addressing the important theme of stress during surgical residency [1]. For a long time surgeons have been known for their indefatigable ‘can-do’ mentality. Demonstrating vulnerability to people outside the surgical community was not something surgeons did. Luckily, more and more attention has been given to stress and burnout, not only among surgical residents but also among surgical attendings. An actual PubMed search with terms [surgeon] AND [burnout] reveals 635 matches. Guglielmetti et all did a nationwide survey among all surgical residents in Switzerland at the time of their yearly examination. The aim of the study was to measure the perceived stress levels of surgical residents, considering sex, age, nationality, and country of graduation. Stress was described as the mental, emotional, or physical reaction caused by internal and external stimuli. Depending on the individual reaction, stress factors may cause positive (eustress) or negative (distress) stress. If a situation is evaluated as overwhelming, uncontrollable, and impossible to cope with, distress inevitably occurs. Chronic stress can lead to burnout: an emotional exhaustion and depersonalization that reduces efficiency at work. Levels of burnout were not considered in the current study.

Over a study period of 5 years, Guglielmetti et al. asked all surgical residents in Switzerland (general, thoracic, cardiac, orthopedics, hand & plastic, pediatric, maxillofacial surgery, and urology) to fill out a questionnaire during their yearly board exam; 1694 (95.7%!!) returned their forms and were included in the current analysis. They used a 10-item questionnaire (PSS-10), which is designed to assess how unpredictable, uncontrollable, and overloaded the respondents value their work lives over the past month. Two subscores are included in the PSS: perceived helplessness and perceived self-efficacy (which has a reverse scale: high value indicates low level of perceived self-efficacy). Perceived helplessness emphasizes the individual’s reaction to stress, while perceived self-efficacy denotes the self-assessed ability to cope with these stressors. 43.5% of the Swiss surgical residents in the present study were women. In their multivariable regression analysis, male sex independently predicted better PSS-10 and PH score. Longer duration of training was an independent predictor for decreasing levels of PSS and PH. Since Switzerland is an immigration country, 37.4% of the Swiss medical workforce did not graduate in Switzerland; those of Italian citizenship and native language or graduating from a country not adjacent to Switzerland reported higher stress and lower self-efficacy. They conclude that perceived stress levels are high in this prospective and representative cohort study of Swiss surgical residents. Females endured significantly worse stress and helplessness levels than males. These figures are worrisome as they may directly contribute to the declining attractivity of surgical residencies. Detailed sex specific analysis and correction of stressors are urgently needed to improve residency programs.

Although Guglielmetti et all don’t go into detail when it comes to the potential causes, they have several suggestions to avoid the perceived stress levels: proper information and transparent expectations for incoming residents on the structure, requirements, and overall organization of the residency programs is one of the easier actions to undertake. Mentorship and physician assistance programs are a further way to improve residency: they can be used to attract medical student towards surgical specialties and, most importantly, to retain residents in training. Structured assistance and counseling aimed at improving work-life integration, supporting family life, increasing access to mental health support, and supporting career transitions have been shows to improve residents’ attrition rate and overall well-being.

Causes for high stress and low self-efficacy are not always clear and often multifactorial; for example, the COVID-19 pandemic has had an enormous negative impact on healthcare workers. In a recent paper in this journal, bullying, discrimination, harassment, and sexual harassment (BDHS) were demonstrated to be often experienced by residents during surgical training, which have been associated with burnout, anxiety, and depression. The majority of residents who experienced BDHS did not report it due to fear of retaliation. Residency programs need to devise methods to have a platform for residents to safely voice complaints and issues [2].

In a cross-sectional national survey of general surgery residents administered with the 2018 American Board of Surgery In-Training Examination, Hu et all assessed mistreatment, burnout and suicidal thoughts during the past year. Weekly burnout symptoms were reported by 38.5% of residents, and 4.5% reported having had suicidal thoughts during the past year. They concluded that mistreatment occurs frequently among general surgery residents, especially for women, and is associated with burnout and suicidal thoughts [3].

So again, we like to congratulate Guglielmetti et all for their timely study of this important topic. We would like to emphasize the great importance of having open discussions with the next generation of surgeons: what are their expectations? How do they want to build their careers? What is their preferred work-life integration? Working across generations of surgeons to assure any BDHS is no longer occurring in surgery, and focusing on ‘energy givers’ instead of ‘energy takers’ will likely reduce stress and burnout among our trainees.
